# Editorial: Young adults or adults who are survivors of childhood cancer: psychosocial side effects, education, and employment

**DOI:** 10.3389/fpsyg.2024.1510822

**Published:** 2024-11-08

**Authors:** Charlotte Demoor-Goldschmidt, Bertrand Porro

**Affiliations:** ^1^Pediatric Oncohematology Department, University Hospital of Angers, Angers, France; ^2^Pediatric Oncohematology Department, University Hospital of Caen, Caen, France; ^3^INSERM U 1018, CESP, Radiation Epidemiology Team, Villejuif, France; ^4^Institut de Cancerologie de l'Ouest (ICO), Angers, France; ^5^Univ Angers, Univ Rennes, Inserm, EHESP, Irset (Institut de recherche en santé, environnement et travail) - UMR_S 1085, SFR ICAT, SIRIC ILIAD, Angers, France

**Keywords:** young adulthood and childhood cancer survivorship, psychosocial side effects, education, employment, resilience

## Introduction

As survival rates for childhood and young adult cancers continue to rise—reaching over 80% for many childhood cancers (Ward et al., 2014)—a growing number of survivors face significant long-term effects on their psychosocial wellbeing, education, and employment. The challenges for survivors extend far beyond remission, with many experiencing cognitive and emotional difficulties that interfere with their quality of life and future opportunities (Armstrong et al., 2009). These effects are compounded as childhood cancer survivors (CCS) and young adult cancer survivors (YACS) transition into adulthood, where they encounter unique barriers related to their cancer history.

This Research Topic, “*Young adulthood and childhood cancer survivorship: psychosocial side effects, education, employment, and resilience*,” brings together critical insights into these challenges. The studies explore how cancer survivors, particularly those who experienced cancer during childhood or early adulthood, manage the psychosocial, educational, and professional aspects of their lives. Additionally, resilience emerges as a key factor in overcoming these challenges, providing a pathway to better long-term outcomes.

## Psychosocial and mental health challenges

The psychosocial difficulties experienced by CCS and YACS are well-documented, with many survivors reporting high levels of emotional distress, anxiety, and depression long after their treatment (Brinkman et al., 2018). In a qualitative study by Ishii and Endo, six Japanese adolescent cancer survivors revealed that managing illness-related uncertainty was a persistent challenge, often manifesting as anxiety about the future. Similarly, Baudry et al., through interviews with 20 French adolescents and young adults (AYA) 5 years post-treatment, found that survivors continue to experience psychological difficulties, including fear of recurrence and social isolation. These findings are consistent with prior research showing that survivors often struggle with mental health long after remission, necessitating ongoing psychosocial support (Zeltzer et al., 2009).

## Education and employment

Educational and vocational challenges are major concerns for both CCS and YACS, as cognitive late effects from cancer treatments, particularly those involving the central nervous system (CNS), can hinder academic and professional success (Armstrong et al., 2010). Ishii and Endo reported that survivors expressed uncertainty about their ability to succeed in education and employment, a concern echoed in Baudry et al.'s study. These findings align with broader research, such as Gurney et al. (2009), which shows that CCS often face barriers in completing education and securing stable employment due to cognitive impairments and physical limitations. Tailored interventions, including individualized learning plans and vocational training, are critical to helping survivors overcome these barriers and achieve their professional goals.

## Parenthood and health concerns

In addition to psychosocial and vocational challenges, many adult survivors of childhood and young adult cancers express concerns about the health of their offspring. Dalkner et al. conducted a quantitative study of 512 participants (256 childhood cancer survivors and 256 siblings), which revealed that many survivors have unfounded fears about passing on genetic predispositions for cancer to their children. This anxiety, despite no evidence of increased cancer risk in their offspring, influences their parenting behaviors. Addressing these concerns through educational interventions and psychological support is essential for helping survivors adopt healthier parenting practices.

## Resilience and long-term outcomes

While the challenges faced by CCS and YACS are significant, resilience has emerged as a key factor in helping survivors manage the long-term effects of their cancer experience. Chen et al. conducted a quantitative cross-sectional study with 286 advanced cancer survivors in China, finding that resilience, supported by social networks and spirituality, significantly improves quality of life (Chen et al., 2023). Although this study focused on adult cancer survivors, its findings on resilience are relevant to CCS, who can similarly benefit from resilience-building strategies to cope with psychosocial, educational, and employment difficulties. Prior studies have also shown that resilience can serve as a buffer against psychological distress, particularly in survivors facing chronic illness (Zebrack et al., 2014).

## Conclusion

The studies within this Research Topic highlight the complex interplay of psychosocial, educational, and vocational challenges that CCS and YACS face as they transition into adulthood. Survivors continue to experience long-term psychological distress, cognitive impairments, and vocational barriers, all of which impact their quality of life. However, resilience emerges as a promising pathway for improving outcomes, with interventions aimed at fostering resilience offering survivors the tools they need to thrive despite their challenges.

To ensure that childhood and young adult cancer survivors not only survive but thrive, comprehensive, long-term support systems must be developed. These systems should incorporate psychological care, educational accommodations, and vocational training, alongside strategies that promote resilience, social support, and emotional wellbeing.
